# Melioidosis in Africa: Time to Uncover the True Disease Load

**DOI:** 10.3390/tropicalmed3020062

**Published:** 2018-06-10

**Authors:** Ivo Steinmetz, Gabriel E. Wagner, Estelle Kanyala, Mamadou Sawadogo, Hema Soumeya, Mekonnen Teferi, Emawayish Andargie, Biruk Yeshitela, Louise Yaba Atsé-Achi, Moussa Sanogo, Bassirou Bonfoh, Raphael Rakotozandrindrainy, Célestin Pongombo Shongo, Mick Shongoya Pongombo, Eric Kasamba Ilunga, Sabine Lichtenegger, Karoline Assig, Jürgen May, Eric Bertherat, Michael Owusu, Ellis Owusu-Dabo, Yaw Adu-Sarkodie

**Affiliations:** 1 Institute of Hygiene, Microbiology and Environmental Medicine, Medical University of Graz, 8036 Graz, Austria; gabriel.wagner-lichtenegger@medunigraz.at (G.E.W.); sabine.lichtenegger@medunigraz.at (S.L.); karoline.assig@medunigraz.at (K.A.); 2 Friedrich Loeffler Institute of Medical Microbiology, University Medicine of Greifswald, KöR, 17475 Greifswald, Germany; 3 Departement UFR/Science de la Santé, Université d’Ouagadougou, BP 7021, Ouagadougou, Burkina Faso; kanyalaestelle@gmail.com (E.K.); elmsawa@yahoo.fr (M.S.); 4 Centre Muraz, 01 BP 390 Bobo Dioulasso, Burkina Faso; meya_4@yahoo.fr; 5 Armauer Hansen Research Institute, Jimma Road, ALERT Compound, P.O. Box 1005 Addis Ababa, Ethiopia; mekonnenteferi@yahoo.com (M.T.); emawaand@gmail.com (E.A.); biruk_23@yahoo.com (B.Y.); 6 Laboratoire Central Vétérinaire de Bingerville, LANADA, P.O. Box 206 Bingerville, Cote D’Ivoire; louisachi@yahoo.fr (L.Y.A.-A.); ssanogomoussas@gmail.com (M.S.); 7 Centre Suisse de Recherches Scientifiques en Côte d’Ivoire (CSRS), 01 BP 1303 Abidjan, Cote D’Ivoire; bassirou.bonfoh@csrs.ci; 8 Department of Microbiology and Parasitology, University of Antananarivo, B.P. 175 Antananarivo, Madagascar; rakrapha13@gmail.com; 9 Université de Lubumbashi, 1825 Lubumbashi, Democratic Republic of the Congo; pongoshon@gmail.com (C.P.S.); mickshongo@yahoo.fr (M.S.P.); kasambailunga@gmail.com (E.K.I.); 10 Bernhard Nocht Institute for Tropical Medicine, 20359 Hamburg, Germany; may@bnitm.de; 11 Department of Infectious Hazard Management, World Health Organization, Geneva 27, Switzerland; bertherate@who.int; 12 College of Health Sciences, Kwame Nkrumah University of Science and Technology, 00233 Kumasi, Ghana; owusumichael-gh@hotmail.com (M.O.); owusudabo@yahoo.com (E.O.-D); yasax@hotmail.co.uk (Y.A.-S.); 13 Kumasi Centre for Collaborative Research, 00233 Kumasi, Ghana

**Keywords:** melioidosis, Africa, Middle East, *Burkholderia pseudomallei*, genomics, public awareness, environment

## Abstract

Melioidosis is an often fatal infectious disease with a protean clinical spectrum, caused by the environmental bacterial pathogen *Burkholderia pseudomallei*. Although the disease has been reported from some African countries in the past, the present epidemiology of melioidosis in Africa is almost entirely unknown. Therefore, the common view that melioidosis is rare in Africa is not evidence-based. A recent study concludes that large parts of Africa are environmentally suitable for *B. pseudomallei*. Twenty-four African countries and three countries in the Middle East were predicted to be endemic, but no cases of melioidosis have been reported yet. In this study, we summarize the present fragmentary knowledge on human and animal melioidosis and environmental *B. pseudomallei* in Africa and the Middle East. We propose that systematic serological studies in man and animals together with environmental investigations on potential *B. pseudomallei* habitats are needed to identify risk areas for melioidosis. This information can subsequently be used to target raising clinical awareness and the implementation of simple laboratory algorithms for the isolation of *B. pseudomallei* from clinical specimens. *B. pseudomallei* was most likely transferred from Asia to the Americas via Africa, which is shown by phylogenetic analyses. More data on the virulence and genomic characteristics of African *B. pseudomallei* isolates will contribute to a better understanding of the global evolution of the pathogen and will also help to assess potential differences in disease prevalence and outcome.

## 1. Introduction

Recently, the global environmental distribution of *B. pseudomallei* and the world-wide incidence and mortality of meliodosis was estimated using a modelling approach. It was predicted that 165,000 melioidosis cases occur per year worldwide, in which 89,000 people die [1]. The estimates suggest not only a massive underreporting in countries known to be endemic but also identified 34 countries in which melioidosis is probably endemic and has never been reported. Among those countries are 24 African countries and three countries in the Middle East. Modelling predicts that 24,000 (95% credible interval 8000–72,000) cases with 15,000 (credible interval 6000–45,000) deaths occur annually in sub-Saharan Africa while less than 1000 annual cases and deaths were predicted for North Africa and the Middle East [1]. Although in some African and Middle East countries sporadic cases of human and animal melioidosis have been reported for many decades [2,3], sound epidemiological data for the disease do not exist for any of those countries. Remarkably, melioidosis has not been classified as a neglected tropical disease. Defining the prevalence of melioidosis in these regions is important for public health. Due to its non-specific clinical presentation, human melioidosis can mimic many common infectious diseases such as malaria or tuberculosis. Due to its severity, misdiagnosis will lead to inappropriate case management and the highest case fatality. Moreover, melioidosis is a differential diagnosis of some epidemic-prone diseases like plague, which means that early diagnosis can be important in a public health perspective. In this paper, we summarize the available information on reported cases of melioidosis in man and animals. Furthermore, we review knowledge on the environmental presence of *B. pseudomallei* in Africa and the Middle East and on the characteristics of African *B. pseudomallei* strains. Lastly, we consider potential strategies to unravel the true burden of the disease in these parts of the world.

## 2. Melioidosis and Environmental *B. pseudomallei* in Africa and the Middle East

In this section, countries were assigned to sub-regions, according to the United Nations geoscheme. Peer-reviewed published cases of human and animal melioidosis together with reports on environmental *B. pseudomallei* in the various African sub-regions and the Middle East (sub-region Western Asia) were compiled based on journal research and by using the global occurrence database created by Limmathurotsakul et al. [1].

### 2.1. Northern Africa

Environmental occurrence of *B. pseudomallei* is associated with high amounts of precipitation [1]. It is, therefore, not surprising that most parts of Northern Africa are not considered suitable environments for this pathogen [1]. Neither indigenous nor imported cases of human melioidosis have been reported from this part of Africa. There is, however, a report from Egypt in 1953 describing melioidosis in a horse. This case was identified by a positive reaction to subcutaneous administration of *Burkholderia mallei* antigen (mallein test) while testing horses for glanders. However, because subsequent antigen tests gave inconsistent results, the authors suspected melioidosis. *B. pseudomallei* was isolated from a mesenteric gland after a post-mortem examination and was shown to be virulent in guinea pigs. Metabolic characteristics of the isolate were different from the close relative *Burkholderia mallei*, indicating that the horse suffered from melioidosis but not glanders [4].

### 2.2. Middle East

The environmental suitability for *B. pseudomallei* seems to vary significantly within the countries of the Middle East [1]. Turkey and parts of the Sinai Peninsula were not predicted to be suitable for *B. pseudomallei*. However, there are regions in Saudi Arabia, Yemen, Iraq, and Oman that seem to be suitable for *B. pseudomallei* [1].

In 1961, a case of pulmonary melioidosis was described in southern Turkey, although the information provided on the bacteriological identification procedure does not allow confirmation of *B. pseudomallei* with certainty [5]. This also applies to a report of *B. pseudomallei* isolation from raw milk samples in different areas of Ankara, Turkey from 1998 [6]. A study from 1997 in Saudi Arabia examined the bacterial flora of the nasal cavity and lungs of healthy and unhealthy sheep and calves. It was reported that *B. pseudomallei* was isolated from the nasal cavity of sheep [7]. Again, the information provided on the bacterial identification procedure does not allow firm conclusions on the identity of the isolated bacteria. In 1997, melioidosis was reported in a camel in Dubai in the United Arab Emirates [8]. Although the clinical signs were suggestive of melioidosis, an inadequate amount of information on microbiological characteristics of the isolated strain was provided [8].

Although large parts of Iran have a relatively dry climate, a subtropical region along the Caspian Sea coast and parts of the south were predicted to be environmentally suitable for *B. pseudomallei* [1]. A report from 1975 described the environmental presence of virulent *B. pseudomallei* in rice fields along the Caspian Sea coast. Out of 157 soil samples, 19 were shown to be positive for *B. pseudomallei* and the virulence of these strains was confirmed in guinea pigs [9]. Furthermore, in 1979, an outbreak of melioidosis among horses and a mule in Iran was reported [10]. The authors isolated motile Gram-negative bacterial strains from abscesses in various organs from the horses and the mule, with biochemical characteristics typical of *B. pseudomallei* but not of *B. mallei* [10]. In 1977, the first case of human pulmonary melioidosis in Iran was published [11]. To our knowledge, there have been no further reports in the international literature of indigenous melioidosis from Iran, except for a case of melioidosis imported from Southeast Asia into Iran [12].

### 2.3. Western Africa

According to the predicted environmental suitability for *B. pseudomallei*, western sub-Saharan Africa is among the highest risk zones [1]. In line with this forecast, *B. pseudomallei* strains were already isolated between 1967 and 1971 from lesions in pigs in a slaughterhouse in Niamey, Niger [13]. Subsequently, environmental surveys along the route of pig transport revealed the presence of *B. pseudomallei* in the soil of Burkina Faso and Niger [14]. More recently, *B. pseudomallei* was isolated in a blood culture of a 59-year-old man native to and living in Burkina Faso, who presented with a mycotic aneurysm of an iliac artery [15]. Galimand and Dodin reported the isolation of *B. pseudomallei* from the soil of a pig farm in Abidjan, Côte d’Ivoire in 1980. However, identification details were not provided [16]. In 1985, the isolation of *B. pseudomallei* in a 12-year-old girl from Sierra Leone presenting with multiple abscesses was reported in The Gambia [17]. In 2009, a 29-year-old diabetic Gambian man was diagnosed in Spain with bilateral calf abscesses due to *B. pseudomallei* after travelling to The Gambia, Guinea-Bissau, and Senegal [18]. Subsequently, another case of melioidosis was reported in a Caucasian Dutch man after traveling to The Gambia. [19].

Among all African countries, Nigeria is predicted to have by far the highest burden of melioidosis [1]. In sharp contrast to this potential disease load, the only report of melioidosis linked to Nigeria was documented in 2011, in which a case of a diabetic traveler was described who most likely acquired infection during a visit to the country [20]. Taken together, the predicted high disease burden and the low number of documented melioidosis incidences exemplify the urgent need for detailed investigations.

In 2014, ‘The African Melioidosis Network’ (AMENET), sponsored in the context of the European Union project ERAfrica, was established. The project aims at serological and environmental surveillance and capacity building in the laboratory diagnosis of *B. pseudomallei*. In West Africa, Burkina Faso, Ghana, and Côte d’Ivoire are among the current target countries. In Côte d’Ivoire, culture screening of lesions in a pig slaughterhouse and some pilot soil sampling did not confirm the presence of *B. pseudomallei* in this country so far [21]. However, a recent report describes cutaneous melioidosis in a 49-year-old man presenting with leg cellulitis and an inguinal abscess after returning from one-year travel in Côte d’Ivoire [22]. Recent environmental qPCR-based direct molecular screening [23] and cultural screening of soil samples in Burkina Faso confirmed the environmental presence of *B. pseudomallei.* The phylogenetic analysis of isolated environmental strains based on whole genome sequencing is under way [24].

### 2.4. Eastern Africa

The predicted environmental suitability for *B. pseudomallei* in Eastern Africa seems to be scattered and possible risk zones vary in size [1]. Among the first hints of melioidosis in this part of the continent was a case of melioidosis in a traveler reported in 1980, who was diagnosed in Denmark but acquired the infection in Kenya. In this case, *B. pseudomallei* identification from blood, urine, and sputum included biochemical characteristics and virulence in guinea pigs [25]. A subsequent environmental survey testing of 81 soil and 71 water samples from three different provinces, including the possible site of infection of the Danish tourist, failed to isolate *B. pseudomallei* [26]. However, recently five indigenous cases of human melioidosis were uncovered in Kenya, even though the overall incidence in this country seems to be low [27]. The only hint of melioidosis in Uganda is a single serological study from 1982 describing 5.9% seropositivity (25 out of 426 individuals) in healthy adults from different parts of the country using an indirect hemagglutination test titre of 1:40 or greater [28]. In 2011, an indigenous case of melioidosis in a child was described in Malawi [2]. Recently, a case of melioidosis in an Eritrean migrant worker was diagnosed in Israel, which indicated that the disease might exist in the Horn of Africa [29].

Parts of Ethiopia were predicted to be environmentally suitable for *B. pseudomallei*. In the context of AMENET, environmental studies revealed the presence of *B. pseudomallei* in soil samples in various regions of the country using molecular [23] as well as cultural methods. The genomes of isolated environmental Ethiopian *B. pseudomallei* strains are currently being analyzed [24]. Current efforts aim to implement serological studies in humans and animals and capacity building in clinical laboratories.

In contrast to the situation in eastern continental Africa, Madagascar, the second largest island state in the western Indian Ocean, was already recognized as an endemic area in the 1930s [30]. Since there is a comprehensive summary of recent cases of melioidosis in the western Indian Ocean [31], in particular in Madagascar, we will only briefly summarize the current situation. First reports on melioidosis in pigs were published by French scientists in 1936 and a further report on the isolation of *B. pseudomallei* from soil [16] confirmed the presence of this pathogen on the island. It took until 2004 for the first human case to be reported and, in 2017, five more cases were described [32]. However, the epidemiological situation for the indigenous population in Madagascar is unknown. Cases were also described from La Réunion, Mauritius, and Seychelles [33,34,35]. In the framework of AMENET, environmental qPCR-based molecular and cultural screening of soil samples was performed and the environmental presence of *B. pseudomallei* could be demonstrated in various regions of Madagascar [36].

### 2.5. Central Africa

*B. pseudomallei* environmental suitability was predicted for all countries of Central Africa. However, significant regional differences seem to exist [1]. The first indication that melioidosis might be present in this African subregion was provided in 1956 by the isolation of *B. pseudomallei* from a lymph node of a goat in Chad [37]. From 2012 to 2013, prospective screening of blood cultures for *B. pseudomallei* was conducted in Gabon and led to the identification of the first melioidosis case in a diabetic female patient in this country [38], and environmental *B. pseudomallei* could be isolated from different sites. In the course of AMENET, the first environmental pilot studies in the DR Congo indicated the presence of *B. pseudomallei* in soil samples [24] by using direct molecular screening, as recently described [23]. Current activities focus on serological surveys and the extension of environmental screening in various subregions. In parallel, clinical surveillance and laboratory screening for human and animal melioidosis is being implemented.

### 2.6. Southern Africa

This sub-region seems to contain relatively limited areas of environmental suitability for *B. pseudomallei* [1]. A report from 1956 described the isolation of *B. pseudomallei* from abscesses of mammary glands and kidneys in a goat from South Africa [39].

## 3. Phenotype, Genomic Diversity, and Phylogenetic Relatedness of *B. pseudomallei* from Africa

At this point in time, the phenotypic characterization of African isolates is essentially restricted to features needed for diagnostic and therapeutic purposes. Wiersinga and colleagues reported that clinical and several environmental features of Gabon isolates showed the typical *B. pseudomallei* diagnostic characteristics, which have also been described in other endemic parts of the world with respect to the API 20NE biochemical profile, colony morphology, antibiotic susceptibility, and *B. pseudomallei*-specific latex agglutination [38]. The known *B. pseudomallei* susceptibility to meropenem, amoxicillin/clavulanic acid, cotrimoxazole and ceftazidime, and characteristic biochemical patterns in respective identification systems, were also reported in isolates from other cases [20,38,40].

As for the phenotypic properties, information on genomic characteristics of *B. pseudomallei* strains from Africa is also based on a limited number of isolates. At this point in time, most available data have been generated using multi-locus sequence typing (MLST), which is a cost-effective and straightforward method for investigating the epidemiology of *B. pseudomallei* [41,42]. The *B. pseudomallei* MLST scheme developed in 2003 is based on seven housekeeping genes and strains are classified according to their allelic profile [43]. Despite the emergence of next generation sequencing (NGS) and its advantages concerning recombination and homoplasy [44,45], MLST still provides valuable insight into the genetic variability of an organism and the geographic distribution/prevalence of different strains, due to its simplicity and sheer amount of deposited STs. As of March 2018, only 26 (0.47%) out of 5494 deposited isolates originate from Africa–compared to 2992 (54.46%) Australian and 906 (16.46%) Thai strains (https://pubmlst.org/bpseudomallei/) [46]. Noteworthily, the African *B. pseudomallei* strains were isolated in as few as eight different countries and do not share a single sequence type (ST) among each other. Furthermore, only four of the 26 STs were also reported in Southeast Asian countries, with none in Australia. While these observations might partially result from the low number of isolates, they already hint at the great genotypic diversity of the African isolates and indicate a segregated, geographically distinct, African subpopulation.

Since the publication of the first closed *B. pseudomallei* genome of the Thai strain K96243 in 2004 [47], the phylogeny of the bacterium has been intensively studied by the emerging NGS methods. At the same time, whole genome sequencing (WGS) offers the advantage of high coverage and, therefore, provides better phylogenetic resolution, which makes it a more robust tool compared to other genetic typing methods that rely on only a few loci [48]. In the first phylogenetic study based on single nucleotide polymorphisms (SNP) of 43 *B. pseudomallei* strains from Asia and Australia, the authors presented strong evidence for an ancestral Australian origin and proposed a single transmission event to Southeast Asia during the glacial periods [45]. Two recent genomic studies, which not only rely on larger datasets but also on more geographically diverse ones, support these findings and further extend them in a global context [49,50]. Phylogenetic analyses show that the South American strains form a sub-cluster within an otherwise African-dominated *B. pseudomallei* clade, which in turn branches from the Asian one [50,51]. Therefore, Sarovich and colleagues hypothesized that *B. pseudomallei* was transmitted from Asia to Africa ~2000 years ago when people from Southeast Asia migrated to Madagascar. Nevertheless, the introduction of *B. pseudomallei* to Africa by birds via the Asia-East Africa flyway is another possibility, which is noted by the authors [49]. Moreover, Chewapreecha et al. estimated the most recent common ancestor for the American strains to be from 1759 or 1806 using the BEAST (Bayesian Evolutionary Analysis by Sampling Trees) method. A time span that coincides with the height of the slave trade (1650–1850) therefore offers a plausible explanation for the onward transmission to the Americas by diseased people or contaminated cargo [50].

It has been argued that the observed difference between predicted and reported meliodosis cases in Africa might not only be due to underreporting and the lack of diagnostic facilities, but it might also be attributed to variations in *B. pseudomallei* virulence [52]. WGS is a well-established technique to address this question on a genetic basis and can further be used to identify geographically-segregated gene variants [50]. For example, a few virulence-associated genes (e.g. *bimA_Bm_*, *fhaB3*) have been described in the literature to correlate with specific clinical presentations and the severity of melioidosis [53]. Therefore, this might account for geographical differences. Additionally, there are reports of African isolates that lack the fimbrial and adhesion virulence protein [52], the type II O-antigenic polysaccharide, or the filamentous hemagglutinin [49]. Even though host and environmental factors are also crucial determinants, it will be most interesting to see how diverse the African *B. pseudomallei* population is and whether bacterial diversity contributes to geographical differences in disease prevalence and outcome. There is no doubt that an extended analysis of more African isolates will not only help to get more detailed insights of *B. pseudomallei* epidemiology and virulence within Africa, but also substantially contributes to our comprehensive understanding of the global evolution and dissemination.

## 4. Current and Future Challenges

In light of the history of melioidosis in other parts of the world, which shows the degree to which highly-endemic regions may go undetected, and the high case fatality rate of undiagnosed melioidosis, there is an urgent need to define risk areas throughout Africa and the Middle East. The first step should be to conduct serological surveys of humans and animals to estimate the degree of exposure to *B. pseudomallei* in various regions. Based on these results, one can then geographically target raising clinical awareness among infectious disease specialists together with microbiological capacity building, which are both needed to detect cases of melioidosis. For the latter, one can learn from other resource-limited settings in which simple laboratory algorithms for the identification of *B. pseudomallei* from clinical specimens have been successfully applied [54]. Known groups at risk such as diabetics will be targeted first.

Serological surveys should be complemented by environmental studies. It will be necessary to systematically determine the environmental distribution and load of *B. pseudomallei* in the different African regions and habitats. When screening soil samples from Thailand and Vietnam, a recent study demonstrated a higher detection rate using a quantitative multi-target PCR approach compared to culture. Moreover, samples with high PCR signals were more likely to be culture-positive compared to samples with low signals [23]. In the environmental studies conducted in Burkina Faso and Madagascar in the context of AMENET, we can confirm that specific molecular signals are obtained at a significantly higher rate when compared to positive cultures.

In endemic regions, prospective studies are then required to determine the incidence and prevalence of melioidosis in humans and animals. Although the limited available information suggests similar clinical presentations and risk factors in African melioidosis cases as compared to other endemic regions, this has to be verified. In this context, it will be important to assess the virulence traits of African *B. pseudomallei* strains compared to Australian and Asian isolates through WGS and experimental infection models.

The identification of environmental factors determining the presence of *B. pseudomallei* and, thereby, the risk of acquiring infection will be important for any prevention and preparedness strategies. Such factors might be identified by e.g., regressing land use patterns, soil types, and vegetation on results from molecular soil screening and seroprevalence data. Studies on *B. pseudomallei* population structure and phylogenetic relatedness offer the chance to analyze past and contemporary environmental dissemination of this pathogen.

The true magnitude of the melioidosis burden in Africa is still to be determined. However, we can expect it to rise in the coming years on this continent like on the others, due to socio-anthropological changes and the increase of the groups at risk for this disease.
